# Association of Peripheral Blood Cell Profile With Alzheimer's Disease: A Meta-Analysis

**DOI:** 10.3389/fnagi.2022.888946

**Published:** 2022-05-06

**Authors:** Le-Tian Huang, Cheng-Pu Zhang, Yi-Bing Wang, Jia-He Wang

**Affiliations:** ^1^Department of Oncology, Shengjing Hospital of China Medical University, Shenyang, China; ^2^Department of Family Medicine, Shengjing Hospital of China Medical University, Shenyang, China; ^3^Department of Urology, Shengjing Hospital of China Medical University, Shenyang, China

**Keywords:** Alzheimer's disease, lymphocyte subsets, meta-analysis, mild cognitive impairment, peripheral blood

## Abstract

**Background:**

Inflammation and immune dysfunction play significant roles in the pathogenesis of Alzheimer's disease (AD)-related dementia. Changes in peripheral blood cell profiles are a common manifestation of inflammation and immune dysfunction and have been reported in patients with AD or mild cognitive impairment (MCI). We systematically evaluated the association of peripheral blood cell counts and indices with AD or MCI through a meta-analysis.

**Methods:**

We electronically searched sources to identify all case–control trials comparing peripheral blood cell counts and/or lymphocyte subsets between patients with AD or MCI and healthy controls (HCs). Meta-analyses were used to estimate the between-group standardized mean difference (SMD) and 95% confidence interval (CI).

**Results:**

A total of 36 studies involving 2,339 AD patients, 608 MCI patients, and 8,352 HCs were included. AD patients had significantly decreased lymphocyte counts (SMD −0.345, 95% CI [−0.545, −0.146], *P* = 0.001) and significantly increased leukocyte counts (0.140 [0.039, 0.241], *P* = 0.006), neutrophil counts (0.309 [0.185, 0.434], *P* = 0.01), and neutrophil–lymphocyte ratio (NLR) (0.644 [0.310, 0.978], *P* < 0.001) compared to HCs. Similarly, significantly increased leukocyte counts (0.392 [0.206, 0.579], *P* < 0.001), NLR (0.579 [0.310, 0.847], *P* < 0.001), and neutrophil counts (0.248 [0.121, 0.376], *P* < 0.001) were found in MCI patients compared with HCs. A significantly decreased percentage of B lymphocytes (−1.511 [−2.775, −0.248], *P* = 0.019) and CD8^+^ T cells (−0.760 [−1.460, −0.061], *P* = 0.033) and a significantly increased CD4/CD8 ratio (0.615 [0.074, 1.156], *P* = 0.026) were observed in AD patients compared to HCs. Furthermore, significant changes in hemoglobin level and platelet distribution width were found in patients with AD or MCI compared with HCs. However, no significant difference was found between AD or MCI patients and HCs in terms of platelet counts, mean corpuscular volume, red cell distribution width, mean platelet volume, and CD4^+^ T, CD3^+^ T, or natural killer cell counts.

**Conclusion:**

Changes in peripheral blood cell profiles, particularly involving leukocyte, lymphocyte, neutrophil, and CD8^+^ T cell counts, as well as the NLR and the CD4/CD8 ratio, are closely associated with AD. The diagnostic relevance of these profiles should be investigated in future.

## Introduction

Alzheimer's disease (AD), a slowly progressive irreversible neurodegenerative disease, is characterized by memory loss of recent events or names as the early clinical symptom; impaired cognition and social function as later symptoms; and dysfunction in speaking, walking, or even swallowing as final symptoms (Reitz and Mayeux, 2014). AD accounts for 60–80% of dementia cases, and its prevalence continues to increase worldwide every year. It is estimated that the number of patients with AD in the USA will grow markedly from 5.8 million in 2020 to 13.8 million by the mid-twenty-first century (Zhang et al., 2021). In 2019, ~$244 billion was spent on AD-related healthcare (Alzheimer's Association, 2021). Given the increasing concern about the aging population, healthcare costs for AD will continue to rise. AD ranks as the fifth-leading cause of death among people of all ages globally (Usman et al., 2021) and has no sufficiently effective treatment options. Unfortunately, numerous clinical trials in patients with AD have failed (Long and Holtzman, 2019). Thus, further studies are needed to understand this disease better.

The diagnostic criteria typically used for AD since 1984 are the National Institute of Neurological and Communicative Disorders and Stroke and the Alzheimer's Disease and Related Disorders Association (NINCDS–ADRDA) criteria, which combine neuropathological patterns and clinical manifestations (Dubois et al., 2007). In 1999, mild cognitive impairment (MCI) was introduced to define an intermediate state between normal cognition and dementia and represents a population at risk of developing AD (Bradfield, 2021).

Extracellular amyloid-β (Aβ) peptide deposition, as neuritic plaques, and intracellular hyperphosphorylated tau (p-tau) protein accumulation, as neurofibrillary tangles, remain key pathological changes in AD and are the primary neuropathological diagnostic criteria for AD (Winblad et al., 2016). Nevertheless, the pathological and clinical manifestations of AD has yet to be elucidated (Long and Holtzman, 2019). There is currently no effective prevention or treatment for Aβ or p-tau accumulation in clinical populations with AD. Other theories, including cholinergic disruption, vascular dysfunction, oxidative stress, immune dysregulation, and neuroinflammation, are emerging as supplementary explanations for this difficult problem (Firdaus and Singh, 2021).

Inflammation and immune dysregulation are involved in the generation of Aβ plaques and neurofibrillary tangles. Initially, the immune response reduces the plaque burden against neurodegeneration through the activation of microglial cells (Sayed et al., 2020). However, with prolonged inflammation, microglial cells lose their ability to clear the plaque (Wyss-Coray et al., 2002; Simard et al., 2006; Sayed et al., 2020). Subsequently, an increasing number of inflammatory cytokines are released, and the number of macrophages increase in an attempt to combat plaque deposition (Stalder et al., 2005; Krabbe et al., 2013). These cytokines and macrophages in turn increase amyloid precursor and p-tau protein levels, resulting in the formation of plaques and neurofibrillary tangles, which exacerbate neural degeneration (Liao et al., 2004; Quintanilla et al., 2004). In this process, the number of peripheral blood cells involved in inflammation and the immune response is increased, including lymphocytes, neutrophils, monocytes, platelets, and lymphocyte subsets (Xue et al., 2009; Chen et al., 2017; Dong et al., 2019; Kara et al., 2022).

Cerebral spinal fluid (CSF) Aβ and p-tau protein have been proven to be biomarkers for identifying older individuals at risk of developing dementia (Langa and Levine, 2014), but they are not widely used because of the difficulty and invasiveness of CSF sampling. Therefore, an easy but feasible way to obtain screening markers, such as peripheral blood cell profile, will be helpful in clinical practice.

Several studies have demonstrated significantly elevated peripheral neutrophil count and/or decreased peripheral lymphocyte count in patients with AD as well as MCI patients compared with age-matched healthy controls (HCs) (Rembach et al., 2014; Chen et al., 2017; Kalelioglu et al., 2017; An et al., 2019; Dong et al., 2019). Some studies have reported an elevated neutrophil-to-lymphocyte ratio (NLR) in those patients (Rembach et al., 2014; Kalelioglu et al., 2017; An et al., 2019; Dong et al., 2019), which has been used to indicate the prognosis of many inflammatory or immune diseases. Changes in the population of other peripheral blood cells were also observed in AD or MCI, including leukocytes, red blood cells (RBCs), and platelets, and their relevant indices such as hemoglobin level, mean corpuscular volume (MCV), red cell distribution width (RDW), mean platelet volume (MPV), and platelet distribution width (PDW) (Chang et al., 2007; Öztürk et al., 2013; Wang et al., 2013; Koç et al., 2014; Liang et al., 2014; Chen et al., 2017; An et al., 2019; Dos Santos and Pardi, 2020). In terms of lymphocyte subsets, several studies have reported an increased percentage of CD4^+^ T cells, decreased percentage of CD8^+^ T cells, and increased CD4/CD8 ratio in patients with AD compared to age-matched HCs (Pirttilä et al., 1992; Shalit et al., 1995; Lombardi et al., 1999; Richartz-Salzburger et al., 2007; Schindowski et al., 2007; Xue et al., 2009; Zhang et al., 2013). Nevertheless, some studies have reported non-significant or even contradictory results. Thus, to date, no consistent conclusion about the changes in peripheral blood cell profiles has been reached.

To better understand the associations between peripheral blood cell profiles and AD related dementia, it is necessary to make a comprehensive meta-analysis. To the best of our knowledge, this study is the first meta-analysis to compare peripheral blood cell counts and indices between patients with AD or MCI and age-matched HCs.

## Methods

### Search Strategy

We systematically searched the PubMed, Cochrane Library, Embase, and Web of Science databases for studies published up to January 20, 2022. The search terms used were: “Alzheimer,” “mild cognitive impairment,” “blood cell count,” “lymphocyte subsets,” “neutrophil,” “eosinophil,” “basophil” “lymphocyte,” “NLR,” “monocyte,” “white blood cell,” “leucocyte,” “red blood cell,” “erythrocyte,” “hemoglobin,” “platelet,” “PLR,” “mean corpuscular volume,” “red cell distribution width,” “mean platelet volume,” “platelet distribution width,” “CD4^+^,” “CD8^+^,” “CD4^+^/CD8^+^,” “CD3^+^,” “B lymphocyte,” and “NK cell.” Relevant conference abstracts and presentations were also searched. Two independent authors screened all articles.

### Selection Criteria

Eligible studies were case–control studies describing the association between peripheral blood cell counts or indices or lymphocyte subsets and AD and/or MCI patients, with data expressed as the mean and standard deviation, and wherein the number of patients was available. Non-comparative studies, duplicated studies, case reports, opinions, editorials, review articles, or studies with insufficient data to allow meta-analysis were excluded.

### Data Collection and Quality Assessment

The following data were collected from the identified studies: authors, publication year, country, disease, number of subjects, median age, diagnostic criteria, Mini-Mental State Examination (MMSE) scores, and peripheral blood cell indices or lymphocyte subsets. The Agency for Healthcare Research and Quality (AHRQ) criteria were used to assess the methodological quality of the included cross-sectional case–control studies, with scores ranging from 0 to 11 points (Chou et al., 2018). Screening of studies, data extraction, and quality assessment were conducted by two researchers independently. Disagreements were resolved by discussion.

### Statistical Analysis

Absolute numbers of peripheral blood cell parameters and percentages of lymphocyte subsets were continuous outcomes, expressed as the standardized mean difference (SMD) with corresponding 95% confidence intervals (CIs). Meta-analysis was performed using STATA software (version 12.0; StataCorp, College Station, TX, USA). In addition, a funnel plot was constructed to evaluate symmetry to visually assess publication bias, and sensitivity analyses were performed for the outcome with the leave-one-out approach to exclude potential bias induced by single study. All tests were two-sided, and statistical significance was set at *P* < 0.05. I-squared statistics were used to assess heterogeneity. I-squared statistic >50% or *P* < 0.10 was defined as significant heterogeneity among studies. The fixed-effects model was used in the absence of heterogeneity, and the random-effects model was used when heterogeneity was present among studies.

## Results

### Study Selection and Characteristics

**Figure 1:**
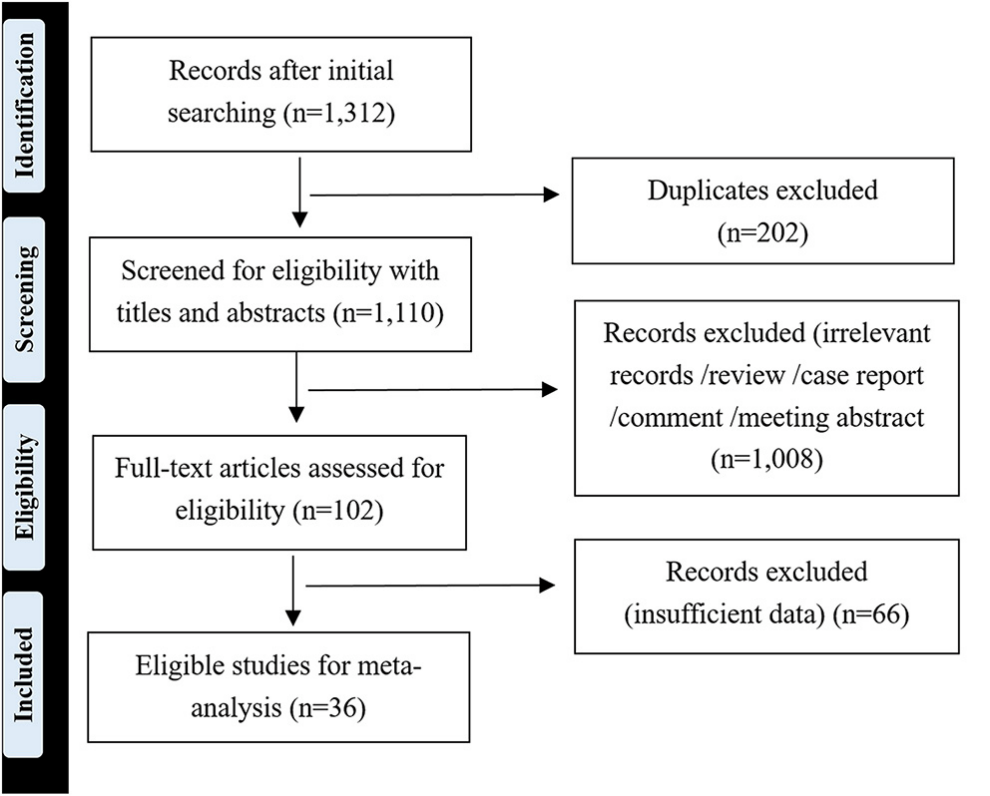
Overview of literature search and selection.

A total of 1,312 publications were screened from databases and conference abstract compilations through an initial database search. After reviewing the titles and abstracts, 102 potential articles were identified and reviewed in detail. Finally, 36 studies were considered to be suitable for our meta-analysis according to the stipulated inclusion and exclusion criteria (Figure 1).

In total, 2,339 AD patients, 608 MCI patients, and 8,352 HCs were identified in the 36 identified studies. Thirty-four studies were included in the comparisons of AD patients and HCs, while six studies compared MCI patients and HCs. These studies were used for meta-analysis of peripheral blood cell counts and relevant indices, including leukocyte count; neutrophil count; NLR, lymphocyte count; monocyte count; RBC count; hemoglobin level; MCV; RDW; platelet count; MPV; PDW; percentage of CD4^+^ T cells, CD8^+^ T cells, CD3^+^ T cells, B lymphocytes, and natural killer (NK) cells; and the CD4/CD8 ratio. Few of these studies had analyzed eosinophils, basophils, MCH, or PLR. The characteristics and quality of the included studies are summarized in Table 1.

**Table 1:** Characteristics of included studies.

**References**	**Country**	**Disease**	**Number of subjects**	**Median age (years)**	**Diagnostic criteria**	**MMSE scores**	**Peripheral blood cell or lymphocyte subset**	Scores*
Kara et al. (2022)	Turkey	AD	AD: 94 HC: 61	AD: 74.2 ± 9.6 HC: 65.7 ± 4.6	DSM-IV	N/A	WBC, N, L, M, PLT, NLR	9
Sun et al. (2022)	China	AD	AD: 127 HC: 100	AD: 67.36 ± 10.2 HC: 68.05 ± 8.73	NINCDS-ADRDA	AD: 11.50 ± 4.56 HC: 27.29 ± 2.09	M	9
Dos Santos and Pardi (2020)	Brazil	AD	AD: 60 HC: 60	AD: N/A HC: ≥60	N/A	N/A	HGB, PLT	8
Du et al. (2020)	China	MCI	MCI: 85 HC: 85	MCI: 70.0 ± 4.77 HC: 70.05 ± 4.73	Peterson's	MCI: 22.07 ± 1.3 HC: 27.25 ± 1.98	HGB, MCV	8
Dong et al. (2019)	China	AD and MCI	AD: 56 MCI: 57 HC: 59	AD: 69.04 ± 9.05 MCI: 70.67 ± 9.26 HC: 68.12 ± 5.81	NINCDS-ADRDA	N/A	WBC, N, L, M, PLT, NLR, RBC, MCV, RDW, MPV, PDW	6
An et al. (2019)	China	MCI	MCI: 186 HC: 153	MCI: 73.10 ± 3.29 HC: 71.19 ± 3.32	N/A	MCI: 20.38 ± 2.13 HC: 23.96 ± 1.59	N, L, NLR, HGB, WBC, PLT	7
Kalelioglu et al. (2017)	Turkey	AD and MCI	AD: 31 MCI: 30 HC: 31	N/A	DSM-IV	N/A	N, L, PLT, NLR	8
Chen et al. (2017)	China	AD	AD: 92 HC: 84	AD: 69.95 ± 10.63 HC: 70.6 ± 5.39	NINCDS-ADRDA	AD: 15.09 ± 4.56 HC: 29.08 ± 1.00	L, RBC, HGB, MPV, MCV, PDW	8
Min and Min (2016)	Korea	AD	AD: 49 HC: 4,639	AD: ≥60 HC: ≥60	N/A	N/A	HGB	6
Rembach et al. (2014)	Australia	AD and MCI	AD: 205 MCI: 130 HC: 759	AD: 78.99 ± 8.4 MCI: 76.25 ± 7.5 HC: 70.57 ± 6.98	AD: NINCDS-ADRDA MCI: Peterson's	AD: 20 ± 5.27 MCI: 26.5 ± 2.66 HC: 29 ± 1.19	N, L, NLR	6
Koç et al. (2014)	Turkey	AD	AD: 109 HC: 81	AD: 76.74 ± 8.99 HC: 75.32 ± 8.42	NINCDS-ADRDA; DSM-IV	AD: 19.27 ± 4.87	PLT, MPV	6
Zhang et al. (2013)	USA	AD	AD: 41 HC: 31	AD: 77.9 ± 7.7 HC: 75.4 ± 9.5	NINCDS-ADRDA	AD: 24.5 ± 2.1	CD4/CD8, CD4^+^%, CD8^+^%	7
Wang et al. (2013)	China	AD and MCI	AD: 120 MCI: 120 HC: 120	AD: 72.8 ± 3.6 MCI: 72.9 ± 3.5 HC: 73.7 ± 4.2	AD: NINCDS-ADRDA MCI: Peterson's	AD: 14.5 ± 2.2 MCI: 24.8 ± 0.8 HC: 27.9 ± 1.5	PLT, MPV, PDW	8
Liang et al. (2014)	China	AD	AD: 110 HC: 150	AD: 73.4 ± 4.0 HC: 72.7 ± 3.9	NINCDS-ADRDA	AD: 15.2 ± 3.1 HC: 27.8 ± 1.6	PLT, MPV, PDW	7
Westman et al. (2013)	Sweden	AD	AD: 50 HC: 50	AD: 77.5 ± 6.9 HC: 74.0 ± 8.0	NINCDS-ADRDA; DSM-IV	AD: 19.9 ± 3.1 HC: N/A	WBC, HGB, L	5
Koçer et al. (2013)	Turkey	AD	AD: 89 HC: 104	AD: 75 (46–88) HC: 72 (60–86)	NINCDS-ADRDA	AD: 18 (6–26) HC: N/A	MPV	5
Öztürk et al. (2013)	Turkey	AD	AD: 197 HC: 133	AD: 76.22 ± 6.92 HC: 71.68 ± 5.3	NINCDS-ADRDA; DSM-IV	AD: 15.79 ± 5.33 HC: 26.75 ± 3.27	WBC, HGB, RDW, PLT	6
Kuyumcu et al. (2012)	Turkey	AD	AD: 241 HC: 175	AD: 76.53 ± 6.00 HC: 71.95 ± 5.40	NINCDS-ADRDA; DSM-IV	AD: 18.32 ± 7.94 HC: 27.08 ± 3.29	WBC, PLT, HGB, NLR	5
Yesil et al. (2012)	Turkey	AD	AD: 126 HC: 286	AD: 76.2 ± 6.8 HC: 75.2 ± 6.3	NINCDS-ADRDA; DSM-IV	AD: 20.1 ± 7.2 HC: 26.0 ± 3.4	WBC, HGB, PLT, MPV	5
Shah et al. (2011)	USA	AD	AD: 113 HC: 768	AD: 85.9 ± 6.3 HC: 80.0 ± 7.4	NINCDS-ADRDA	AD: 25.9 ± 2.6 HC: 28.2 ± 1.8	HGB, MCV, RDW	6
Xue et al. (2009)	China	AD	AD: 48 HC: 30	AD: 74.5 ± 9.8 HC: 71.7 ± 8.9	NINCDS-ADRDA; DSM-IV	AD: 17.2 HC: 29.8	CD3^+^%, CD4^+^%, CD8^+^%, B lymphocyte%, NK cell%	8
Larbi et al. (2009)	Canada	AD	AD: 12 HC: 6	AD: 75.4 ± 7.1 HC: 74.0 ± 3.8	NINCDS-ADRDA; DSM-IV	AD: 25 HC: 30	WBC, HGB, PLT	7
Bonotis et al. (2008)	Greece	AD	AD: 23 HC: 21	AD: 76.35 ± 6.9 HC: 71.23 ± 4.4	NINCDS-ADRDA	AD: 19.76 ± 2.2 HC: 28.33 ± 1.3	CD4^+^%, CD8^+^%, CD4/CD8	5
Speciale et al. (2007)	Italy	AD	AD: 51 HC: 51	AD: 72.2 ± 8.8 HC: 69.1 ± 8.2	NINCDS-ADRDA	AD: 17.57 ± 6.19 HC: 28.5 ± 1.03	B lymphocyte%	7
Richartz-Salzburger et al. (2007)	Germany	AD	AD: 43 HC: 34	AD: 70.9 ± 8.2 HC: 67.5 ± 7.3	NINCDS-ADRDA	AD: 17.9 HC: N/A	CD3^+^%, CD4^+^%, CD8^+^%, CD4/CD8, B lymphocyte%, NK cell%	6
Schindowski et al. (2007)	Germany	AD	AD: 24 HC: 34	AD: 73.4 ± 3.5 HC: 71.5 ± 4.6	NINCDS-ADRDA	AD: 18.8 ± 1.12 HC: N/A	CD4^+^%, CD8^+^%, CD4/CD8	5
Chang et al. (2007)	China	AD	AD: 21 HC: 23	AD: 76 ± 3 HC: 77 ± 4	NINCDS-ADRDA; DSM-IV	AD: 24.1 ± 0.4 HC: 28.9 ± 0.9	WBC, RBC, HGB, MCV, PLT	6
Schindowski et al. (2006)	Germany	AD	AD: 23 HC: 25	AD: 72.2 ± 3.1 HC: 74.0 ± 4.9	NINCDS-ADRDA	AD: 19.1 ± 1.48 HC: N/A	CD3^+^%, B lymphocyte%, NK cell%	8
Armanini et al. (2003)	Italy	AD	AD: 23 HC: 23	AD: 67 (49–89) HC: 66 (49–89)	NINCDS-ADRDA; DSM-IV	AD: 20 (15–24) HC: N/A	CD4^+^%, CD8^+^%, CD4/CD8	6
Lombardi et al. (1999)	Spain	AD	AD: 45 HC: 45	AD: 69 (63–76) HC: 71 (61–79)	NINCDS-ADRDA; DSM-III-R	N/A	CD3^+^%, CD4^+^%, CD8^+^%, NK cell%	8
Song et al. (1999)	Belgium	AD	AD: 15 HC: 16	AD: 78.4 ± 10.3 HC: 75.3 ± 8.8	DSM-IV	AD: 8.0 ± 6.7 HC: N/A	L, N, WBC, M	5
Shalit et al. (1995)	Israel	AD	AD: 12 HC: 13	AD: 76.2 ± 7.4 HC: 75.2 ± 6.1	NINCDS-ADRDA	AD: 15.7 ± 1.97 HC: 28.85 ± 0.39	L, CD4^+^%, CD8^+^%	5
Inestrosa et al. (1993)	Chile	AD	AD: 18 HC: 32	AD: 69.2 ± 1.5 HC: 67.8 ± 1.4	NINCDS-ADRDA	N/A	PLT	5
Pirttilä et al. (1992)	Finland	AD	AD: 33 HC: 35	AD: 65.5 ± 6.9 HC: 61.8 ± 6.8	NINCDS-ADRDA	N/A	L, CD4^+^%, CD8^+^%, CD4/CD8, B lymphocyte%	6
Ikeda et al. (1991)	Japan	AD	AD: 13 HC: 13	AD: 61 ± 8 HC: 57 ± 7	NINCDS-ADRDA; DSM-III-R	N/A	CD4^+^%, CD8^+^%, CD4/CD8	5
Araga et al. (1990)	Japan	AD	AD: 25 HC: 22	AD: 78.0 (59–93) HC: 77.9 (65–88)	DSM-III-R	N/A	CD4^+^%, CD8^+^%, CD4/CD8	6

*Agency for Healthcare Research and Quality (AHRQ) scores for assessing the methodological quality of included studies. AD, Alzheimer's disease; DSM, Diagnostic and Statistical Manual of Mental Disorder; HC, healthy control; HGB, hemoglobin; L, lymphocyte; M, monocyte; MCI, mild cognitive impairment; MCV, mean corpuscular volume; MMSE, Mini Mental State Examination; MPV, mean platelet volume; N, neutrophil; N/A, not applicable; NINCDS–ADRDA, National Institute of Neurologic and Communicative Disorders and Stroke and the Alzheimer's Disease and Related Disorders Association; NK cell, natural killer cell; NLR, neutrophil-lymphocyte ratio; PDW, platelet distribution width; PLT, platelet; RBC, red blood cell; RDW, red cell distribution width; WBC, white blood cell (leucocyte).

### Association of Peripheral Leucocytes With AD or MCI Patients

Total leukocyte counts were first analyzed, and then the subclasses of leukocytes were explored, including polymorphonuclear cells (PMNs) and peripheral blood mononuclear cells (PBMCs). The meta-analysis results are presented in Table 2.

**Table 2:** Summary of meta-analyses.

				**Effect estimates**		**Heterogeneity estimates**		
**Blood indices**	**Number of studies**	**Patients with AD or MCI**	**Patients with HC**	**SMD [95% CI]**	***P*-value**	**I-squared**	***P*-value**	**Model**
**AD vs. HC**								
Leucocyte	9	812	809	0.140 [0.039, 0.241]	0.006	46.20%	0.062	Fixed
Neutrophil	5	401	926	0.309 [0.185, 0.434]	<0.001	0.00%	0.712	Fixed
NLR	5	627	1,085	0.644 [0.310, 0.978]	<0.001	86.50%	<0.001	Random
Lymphocyte	9	588	1,108	−0.345 [−0.545, −0.146]	0.001	59.60%	0.011	Random
Monocyte	4	292	236	−0.318 [−0.707, 0.072]	0.11	76.30%	0.005	Random
CD4^+^ T cell%	11	328	301	0.468 [−0.166, 1.102]	0.148	92.70%	<0.001	Random
CD8^+^ T cell%	11	328	301	−0.760 [−1.460, −0.061]	0.033	93.70%	<0.001	Random
CD4/CD8	8	223	213	0.615 [0.074, 1.156]	0.026	86.20%	<0.001	Random
CD3^+^ T cell%	4	159	134	−1.763 [−4.405, 0.879]	0.191	98.50%	<0.001	Random
B Lymphocyte%	5	174	175	−1.511 [−2.775, −0.248]	0.019	96.00%	<0.001	Random
NK cell%	4	159	134	−0.111 [−1.149, 0.927]	0.834	94.50%	<0.001	Random
RBC	3	169	166	−0.520 [−1.289, 0.250]	0.186	90.50%	<0.001	Random
Hemoglobin	10	961	6,224	−0.347 [−0.563, −0.131]	0.002	81.10%	<0.001	Random
MCV	4	282	934	0.213 [−0.197, 0.623]	0.309	83.50%	<0.001	Random
RDW	3	366	960	0.250 [−0.200, 0.700]	0.276	89.60%	<0.001	Random
Platelet	13	1195	1,217	0.071 [−0.175, 0.318]	0.57	86.90%	<0.001	Random
MPV	7	702	884	−0.247 [−0.988, 0.495]	0.514	97.90%	<0.001	Random
PDW	4	378	413	−1.195 [−1.796, −0.595]	<0.001	93.20%	<0.001	Random
**MCI vs. HC**								
Leucocyte	2	243	212	0.392 [0.206, 0.579]	<0.001	46.20%	0.173	Fixed
Neutrophil	4	403	1,002	0.248 [0.121, 0.376]	<0.001	26.10%	0.255	Fixed
NLR	4	403	1,002	0.579 [0.310, 0.847]	<0.001	70.80%	0.016	Random
Lymphocyte	4	403	1,002	−0.209 [−0.515, 0.096]	0.179	78.10%	0.003	Random
Hemoglobin	2	271	238	−0.869 [−1.927, 0.189]	0.107	96.40%	<0.001	Random
MCV	2	141	144	−0.104 [−0.441, 0.234]	0.546	50.90%	0.153	Random
Platelet	4	393	363	−0.073 [−0.217, 0.070]	0.315	47.10%	0.129	Fixed
MPV	2	176	179	−0.342 [−1.899, 1.215]	0.667	97.80%	<0.001	Random
PDW	2	176	179	−0.446 [−1.485, 0.593]	0.4	95.20%	<0.001	Random

*AD, Alzheimer's disease; CI, confidence interval; HC, healthy control; MCI, mild cognitive impairment; MCV, mean corpuscular volume; MPV, mean platelet volume; NK cells, natural killer cells; NLR, neutrophil-lymphocyte ratio; PDW, platelet distribution width; RBC, red blood cell; RDW, red cell distribution width; SMD, standardized mean difference*.

**Figure 2:**
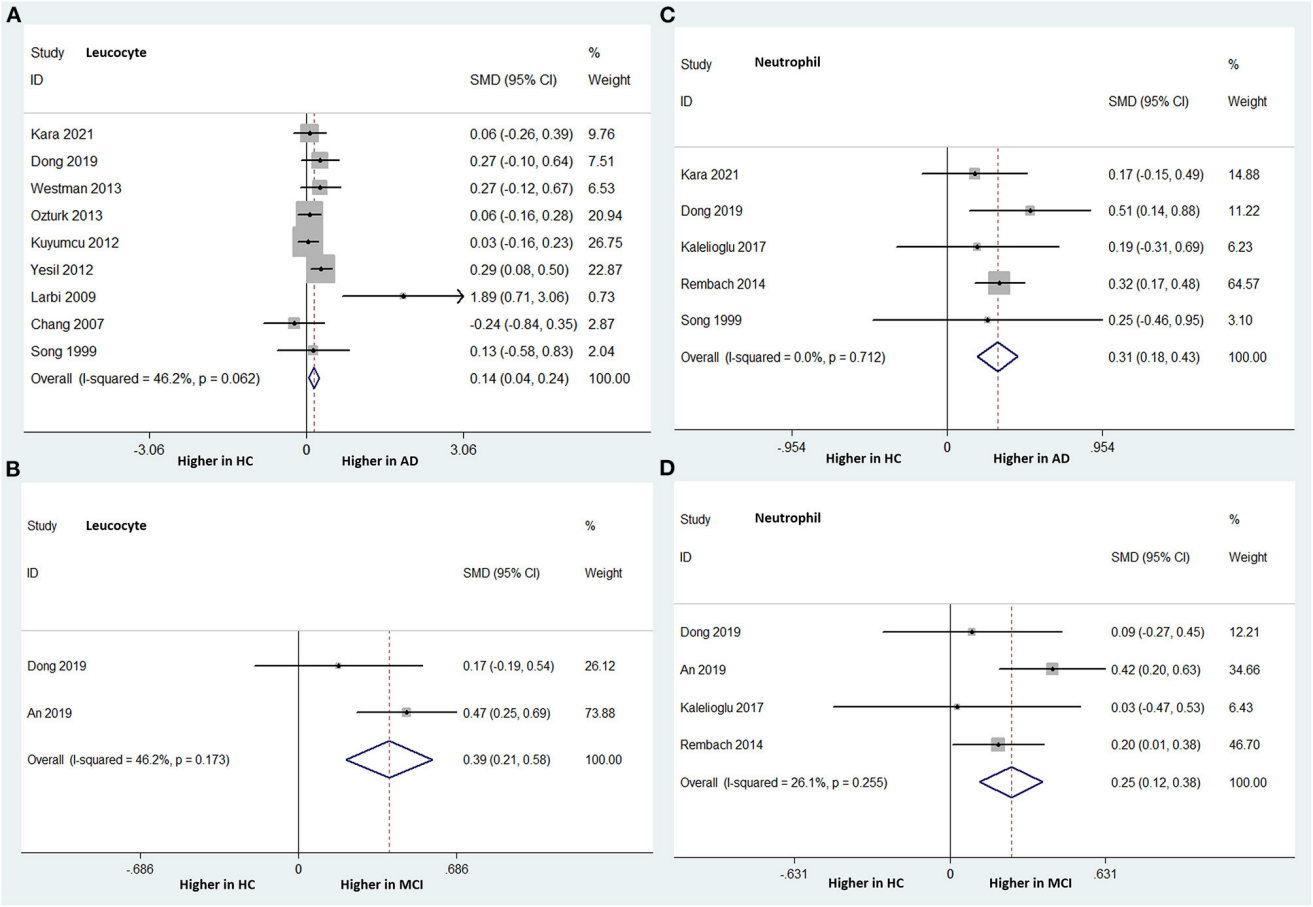
Forest plots of standardized mean differences (SMDs) for leucocyte counts in Alzheimer's disease (AD) **(A)** and mild cognitive impairment (MCI) **(B)**, neutrophil counts in AD **(C)** and MCI **(D)**, compared to healthy controls (HCs).

Nine studies (involving 1,621 subjects) compared the peripheral leukocyte count between patients with AD and HCs. AD patients had a significantly higher leukocyte count than HCs (SMD: 0.140; 95% CI: 0.039–0.241; *P* = 0.006), without significant heterogeneity among the studies (I-squared = 46.2%, *P* = 0.062) (Figure 2A). Two studies (involving 455 subjects) analyzed the peripheral leukocyte count differences between MCI patients and HCs. The peripheral leukocyte counts were significantly higher in the MCI group than in the HC group. The overall pooled SMD was 0.392 (95% CI: 0.206–0.579, *P* < 0.001). There was no significant heterogeneity among the studies (I-squared = 46.2%, PP = 0.173) (Figure 2B).

#### PMNs

PMNs include neutrophils, eosinophils, and basophils. However, only neutrophils were analyzed in several studies. The NLR was also analyzed.

##### Neutrophil Count

Five studies (involving 1,327 subjects) analyzed the peripheral neutrophil count differences between AD patients and HCs. The AD group had a significantly higher peripheral neutrophil count than did the HC group. The overall pooled SMD was 0.309 (95% CI: 0.185–0.434, *P* < 0.001). There was no significant heterogeneity among the studies (I-squared = 0.0%, *P* = 0.712) (Figure 2C).

Four studies (involving 1,405 subjects) analyzed the peripheral neutrophil count differences between patients with MCI and HCs. The peripheral neutrophil count was statistically significantly higher in the MCI patients than in the HC group. The overall pooled SMD was 0.248 (95% CI: 0.121–0.376, *P* < 0.001). There was no significant heterogeneity among the studies (I-squared = 26.1%, *P* = 0.255) (Figure 2D).

##### NLR

**Figure 3:**
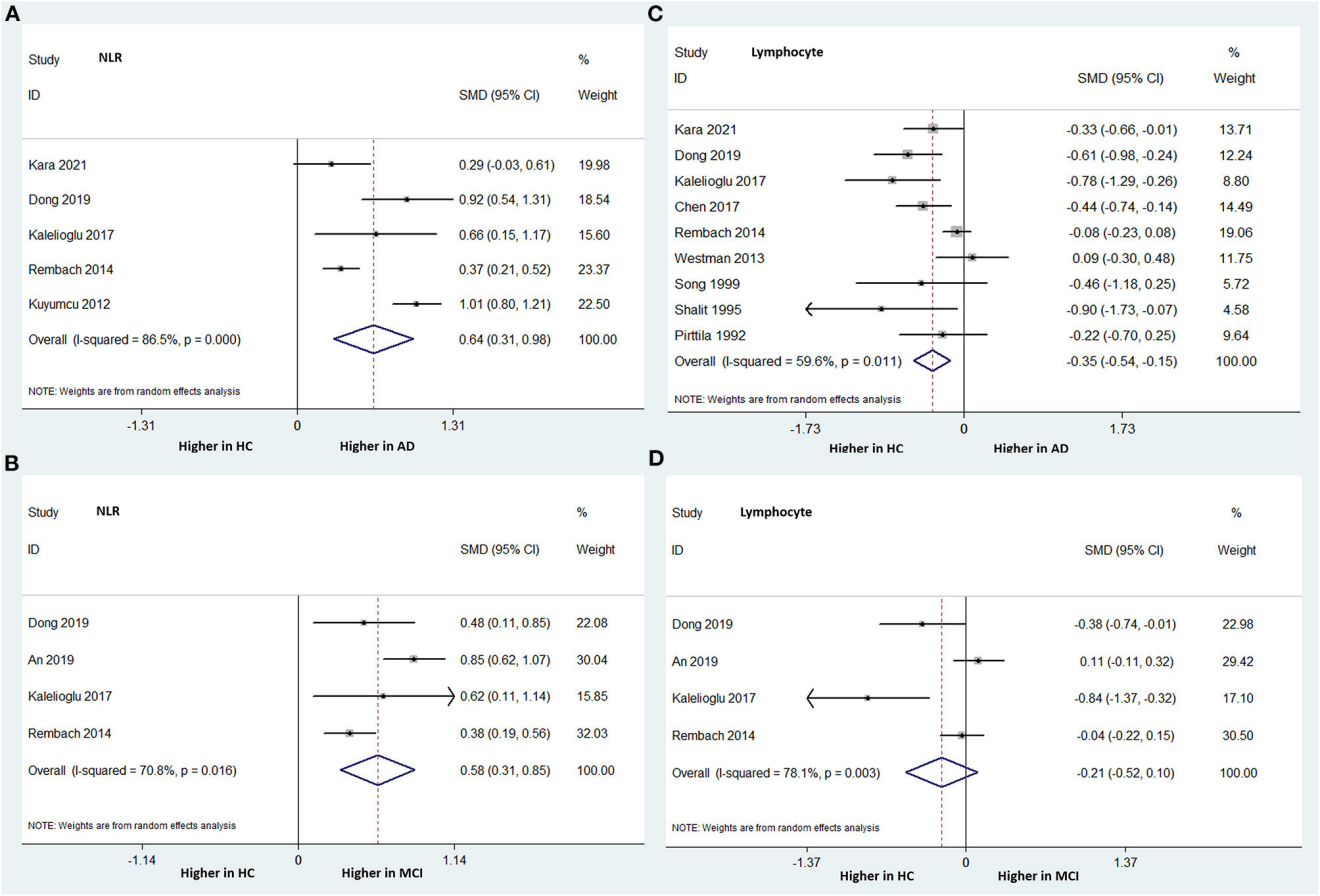
Forest plots of standardized mean differences (SMDs) for the neutrophil–lymphocyte ratio (NLR) in Alzheimer's disease (AD) **(A)** and mild cognitive impairment (MCI) **(B)**, and lymphocyte counts in AD **(C)** and MCI **(D)**, compared to healthy controls (HCs).

Five studies, including 1,712 subjects, analyzed the NLR differences between AD patients and HCs. The NLR was statistically significantly higher in the AD group than in the HC group. The overall pooled SMD was 0.644 (95% CI: 0.310–0.978, *P* < 0.001), with significant heterogeneity among the studies (I-squared = 86.5%, *P* < 0.000) (Figure 3A).

Four studies, including 1,405 subjects, compared the NLR between MCI patients and HCs. Similarly, the MCI group had a significantly higher NLR than the HC group. The overall pooled SMD was 0.579 (95% CI: 0.310–0.847, *P* < 0.001), with significant heterogeneity among the studies (I-squared = 70.8%, *P* = 0.016) (Figure 3B).

#### PBMCs

The total PBMC count, including lymphocytes, monocytes, T cells with different surface antigens, B lymphocytes, and NK cells, was analyzed to compare the differences between patients with AD or MCI and HCs.

##### Lymphocyte Count

Nine studies (involving 1,696 subjects) analyzed the peripheral lymphocyte count differences between patients with AD and HCs. The AD group had significantly lower peripheral lymphocyte counts than did the HC group. The overall pooled SMD was −0.345 (95% CI: −0.545 to −0.146, *P* = 0.001) (Figure 3C). There was significant heterogeneity among the studies (I-squared = 59.6%, *P* = 0.011).

Four studies (involving 1,405 subjects) compared the peripheral lymphocyte counts between patients with MCI and HCs. The peripheral lymphocyte count was relatively lower in the MCI group than in the HC group; however, the difference was not statistically significant. The overall pooled SMD was −0.209 (95% CI: −0.515–0.096, *P* = 0.179) (Figure 3D). There was significant heterogeneity among the studies (I-squared = 78.1%, *P* = 0.003).

##### CD4^+^ and CD8^+^ T Cell Percentages and CD4/CD8 Ratio

**Figure 4:**
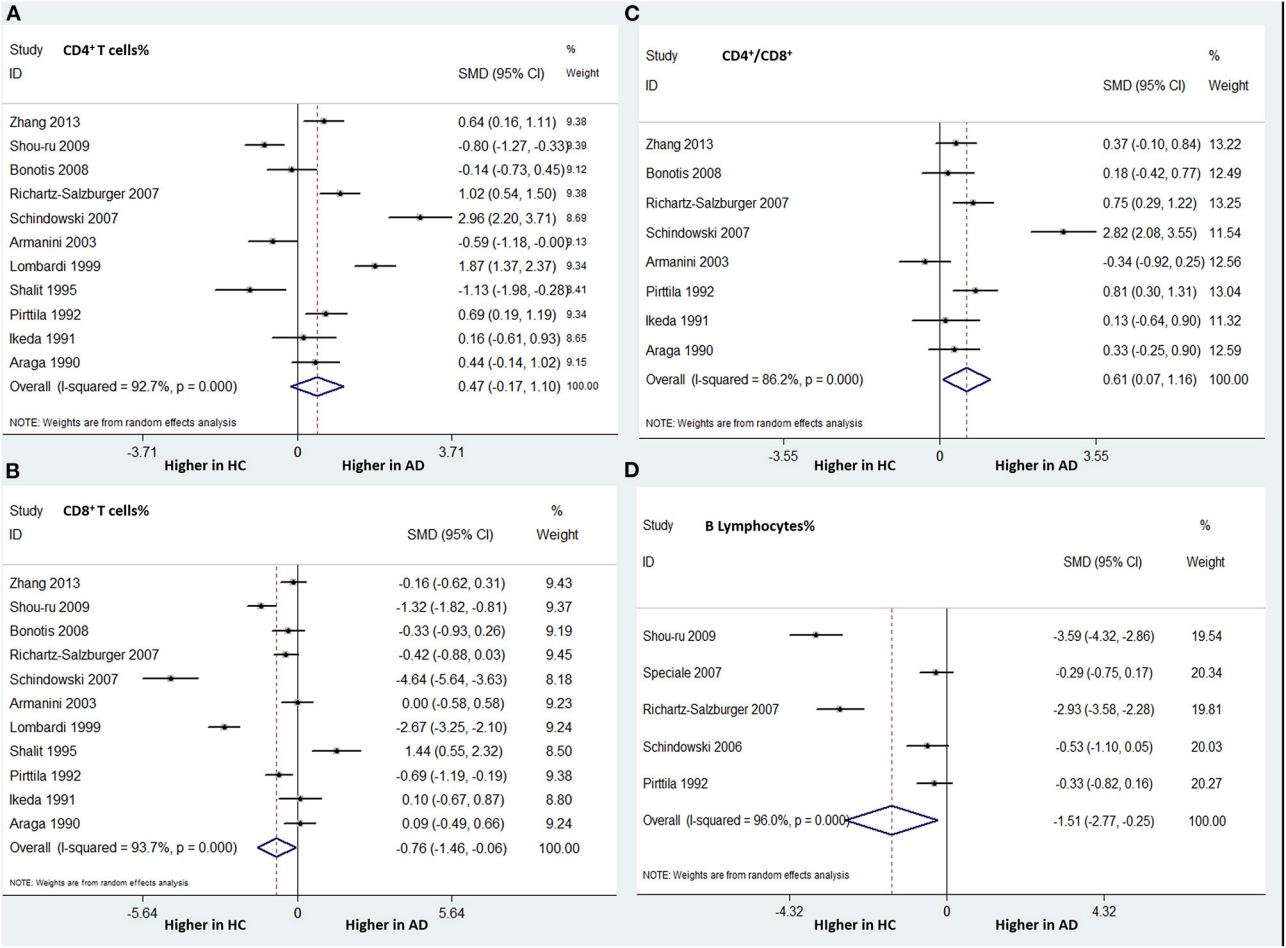
Forest plots of standardized mean differences (SMDs) for percentage of CD4^+^ T cells **(A)** and CD8^+^ T cells **(B)**, the CD4/CD8 ratio **(C)**, and percentage of B lymphocytes **(D)** in Alzheimer's disease (AD), compared to healthy control (HCs).

Eleven studies (involving 328 patients with AD and 301 HCs) analyzed the CD4^+^ T cell percentage differences. There was no statistically significant difference between the two groups in this parameter (SMD: 0.144; 95% CI: −0.657–0.945; *P* = 0.724) (Figure 4A).

The same 11 studies compared the CD8^+^ T cell percentages between patients with AD and HCs. A significantly lower percentage of CD8^+^ T cells was found in patients with AD than in HCs (SMD: −0.760; 95% CI: −1.460 to −0.061; *P* = 0.033). There was significant heterogeneity among these studies (I-squared = 93.7%, *P* < 0.001) (Figure 4B).

Eight studies (involving 436 patients) compared the CD4/CD8 ratios between AD patients and HCs. The AD group had a significantly higher CD4/CD8 ratio than did the HC group (SMD: 0.615; 95% CI: 0.074–1.156; *P* = 0.026), with significant heterogeneity (I-squared = 86.2%, *P* < 0.001) (Figure 4C).

The number of studies on these indices between patients with MCI and HCs was insufficient to perform a meta-analysis.

##### B, NK, and CD3^+^ T Cell Percentages

Five studies (involving 349 subjects) analyzed the differences in the percentage of B lymphocytes between patients with AD and HCs. We found that the AD group had a significantly lower percentage of B lymphocytes than did the HC group. The overall pooled SMD was −1.511 (95% CI: −2.775 to −0.248, *P* = 0.019) (Figure 4D). There was significant heterogeneity among the studies (I-squared = 96.0%, *P* < 0.001).

There was no significant difference in the CD3+ T cell or NK cell percentage between patients with AD and HCs. The number of studies on these indices between patients with MCI and HCs was insufficient to perform a meta-analysis.

##### Monocyte Count

Four studies (involving 528 subjects) compared peripheral monocyte counts between patients with AD and HCs. The AD group had a relatively lower peripheral monocyte count than did the HC group; however, the difference was not statistically significant. The overall pooled SMD was −0.318 (95% CI: −0.707–0.072, *P* = 0.179). There was significant heterogeneity among the studies (I-squared = 76.3%, *P* = 0.005).

The number of studies on peripheral monocyte count differences between MCI patients and HCs was insufficient to allow meta-analysis.

### Association of Peripheral RBCs With AD or MCI Patients

Total RBC counts and correlated indices, such as hemoglobin level, RDW, and MCV, were analyzed. The meta-analysis results are presented in Table 2.

#### RBC Counts

Three studies (involving 335 subjects) compared the peripheral RBC counts between patients with AD and HCs. The AD group had a relatively lower RBC count than did the HC group; however, the difference was not statistically significant. The overall pooled SMD was −0.520 (95% CI: −1.289 to 0.250, *P* = 0.186). There was significant heterogeneity among these studies (I-squared = 90.5%, *P* < 0.001). Few studies compared the RBC counts between MCI and HC groups.

#### Hemoglobin, RDW, and MCV

**Figure 5:**
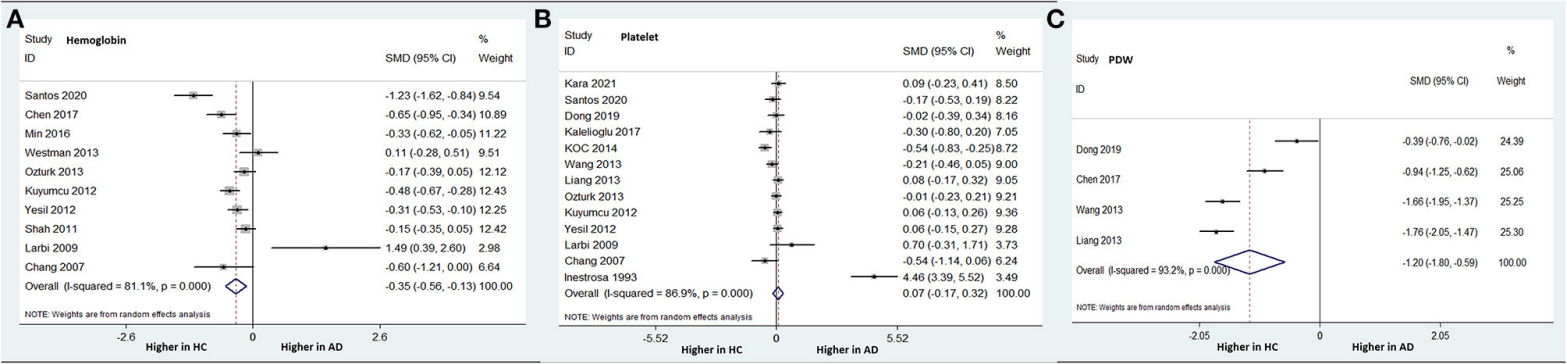
Forest plots of standardized mean differences (SMDs) for hemoglobin level **(A)**, platelet count **(B)**, and platelet distribution width (PDW) **(C)** in Alzheimer's disease (AD) compared to healthy controls (HCs).

Ten studies (involving 961 patients with AD and 6,224 HCs) compared the peripheral hemoglobin levels between AD patients and HCs. In contrast to the RBC results, patients with AD had significantly lower hemoglobin levels than did HCs (SMD −0.347; 95% CI: −0.563 to −0.131; *P* = 0.002) (Figure 5A). There was significant heterogeneity among the studies (I-squared = 81.1%, *P* < 0.001). However, no difference was found in hemoglobin levels between patients with MCI and HCs.

There was no significant difference regarding RDW and MCV between the AD or MCI and HC groups.

### Platelets in AD or MCI Patients

Platelet counts and correlated indices, such as PDW and MPV, were analyzed. The meta-analysis results are presented in Table 2.

#### Platelet Count

Thirteen studies were included in the comparison of peripheral platelet count between patients with AD and HCs, but no difference was found between the two groups (SMD 0.071; 95% CI: −0.175–0.318; *P* = 0.57) (Figure 5B). Additionally, there was no difference in the platelet counts between patients with MCI and HCs.

#### PDW and MPV

Four studies (involving 791 subjects) compared the peripheral PDW between AD patients and HCs. Patients with AD had a significantly lower PDW than did HCs (SMD −1.195; 95% CI: −1.796 to −0.595; *P* < 0.001) (Figure 5C). There was significant heterogeneity among the studies (I-squared = 93.2%, *P* < 0.000). There was no difference in PDWs between patients with MCI and HCs.

Furthermore, there was no significant difference between the AD, MCI, and HC groups regarding the MPV.

### Subgroup Analyses Based on Region

Based on the region of population in these studies, we divided them into Asian and European. Subgroup meta-analyses of peripheral blood cell comparisons were performed when there were at least two studies of one region. Minor differences were found in the results (Table 3).

**Table 3:** Subgroup meta-analyses based on region.

	**European**			**Asian**			**Total**		
**Blood indices**	**Number of studies**	**SMD [95% CI]**	***P*-value**	**Number of studies**	**SMD [95% CI]**	***P*-value**	**Number of studies**	**SMD [95% CI]**	***P*-value**
**AD vs. HC**									
Leucocyte	7	0.141 [0.035, 0.248]	0.009	2	0.128 [−0.185, 0.440]	0.423	9	0.140 [0.039, 0.241]	0.006
Lymphocyte	7	−0.279 [−0.507, −0.052]	0.016	2	−0.505 [−0.739, −0.271]	0.000	9	−0.345 [−0.545, −0.146]	0.001
Monocyte	2	−0.129 [−1.288, 1.030]	0.827	2	−0.379 [−0.829, 0.070]	0.098	4	−0.318 [−0.707, 0.072]	0.11
CD4^+^ T cell%	8	0.672 [−0.088, 1.432]	0.083	3	−0.091 [−0.922, 0.739]	0.829	11	0.468 [−0.166, 1.102]	0.148
CD8^+^ T cell%	8	−0.908 [−1.830, 0.015]	0.054	3	−0.396 [−1.399, 0.607]	0.439	11	−0.760 [−1.460, −0.061]	0.033
CD4/CD8	6	0.740 [0.047, 1.432]	0.036	2	0.257 [−0.205, 0.719]	0.275	8	0.615 [0.074, 1.156]	0.026
Hemoglobin	6	−0.166 [−0.382, 0.051]	0.134	3	−0.496 [−0.714, −0.278]	0.000	10	−0.347 [−0.563, −0.131]	0.002
Platelet	7	−0.054 [−0.243, 0.135]	0.576	4	−0.102 [−0.310, 0.106]	0.337	13	0.071 [−0.175, 0.318]	0.570
MPV	3	0.247 [−0.263, 0.758]	0.342	4	−0.619 [−1.831, 0.592]	0.316	7	−0.247 [−0.988, 0.495]	0.514
**MCI vs. HC**									
Neutrophil	2	0.176 [0.002, 0.351]	0.048	2	0.248 [0.121, 0.376]	<0.001	4	0.330 [0.144, 0.516]	<0.001
NLR	2	0.404 [0.229, 0.580]	<0.001	2	0.692 [0.337, 1.047]	<0.001	4	0.579 [0.310, 0.847]	<0.001
Lymphocyte	2	−0.400 [−1.185, 0.385]	0.318	2	−0.110 [−0.580, 0.361]	0.648	4	−0.209 [−0.515, 0.096]	0.179

*AD, Alzheimer's disease; CI, confidence interval; HC, healthy control; MCI, mild cognitive impairment; MPV, mean platelet volume; NLR, neutrophil-lymphocyte ratio; SMD, standardized mean difference*.

### Study Quality, Publication Bias, and Sensitivity Analyses

We assessed the methodological limitations of all the included studies using the AHRQ scale. All studies scored more than 5, indicating that these studies presented moderate or high-quality results (Table 1). A low publication bias was observed in the funnel plot. Sensitivity analysis for each peripheral blood cell comparison between AD and HCs was performed, and no significant heterogeneity from any study was found (Supplementary File 1).

## Discussion

To gain a better understanding of the association of circulating inflammatory and immune blood cell profiles with the severity of AD-related dementia, we here performed a meta-analysis on 36 studies that included a total of 2,339 AD patients, 608 MCI patients, and 8,352 HCs, comparing not only AD patients and HCs but also MCI patients and HCs. To date, no meta-analysis on this topic has been conducted. According to our data, compared to HCs, patients with AD had significantly increased neutrophil, leukocyte, and CD4^+^/CD8^+^ T cell counts and NLR, as well as decreased lymphocyte counts, hemoglobin levels, PDW, and percentage of CD8^+^ T cells and B lymphocytes. Furthermore, significantly elevated neutrophil and leukocyte counts and NLR were observed in MCI patients compared with HCs. Our results suggest that significant alterations in peripheral inflammatory cells and lymphocyte subsets may be associated with the pathogenesis of AD and MCI.

Significant alterations in the population of neutrophils, leukocytes, and lymphocytes and NLR in AD patients, reflecting the body's inflammation, stress, and immune response, suggest that lymphocyte and neutrophil proliferation may be influenced by pathophysiological mechanisms of AD, including oxidative stress reactions, immune dysregulation, and neuroinflammation (Pluta et al., 2018). As key inflammatory cells, neutrophils can increase in number by some inflammatory cytokines, such as tumor necrosis factor-alpha (TNF-α) and interleukin-9, which are involved in the pathogenesis of AD (Cowburn et al., 2004; Holmes et al., 2011). Baj et al. found that chronic release of TNF-α by microglia could lead to the accumulation of Aβ and p-tau (Baj and Seth, 2018). Increased neutrophil counts, in turn, stimulate T cells through upregulation of antigen presentation, resulting in increased activation of neutrophils and release of TNF-α in a bi-directional manner (Dong et al., 2019). Moreover, activated neutrophils exacerbate neurodegenerative diseases by damaging the blood–brain barrier (BBB). Thus, neuroinflammation is exacerbated as increasing numbers of inflammatory cells and cytokines migrate across the compromised BBB (Baik et al., 2014; Pietronigro et al., 2017; Sayed et al., 2020). With more peripheral neutrophils and lymphocytes migrating into the central nervous system and with elevated neutrophil production in the circulation, the peripheral NLR is elevated significantly in patients with AD and MCI. According to our results, similar changes in the neutrophil count and NLR were also found in MCI patients compared to HCs, but there was no significant decrease in lymphocyte count in the former. This result might represent the slow progressive nature of the neurodegenerative disease. We did not perform a direct comparison between AD and MCI patients because of the limited number of relevant studies. Future research on the different grades of AD-related dementia is required.

Earlier case–control studies on lymphocyte subsets delivered conflicting results, and no consistent conclusion has been reached regarding lymphocyte subset distribution. In the present meta-analysis, we confirmed the increased peripheral CD4/CD8 ratio as well as the decreased percentage of CD8^+^ T cells and B lymphocytes in patients with AD compared to HCs. Decreased CD8^+^ T cell percentage was found in patients with AD but not in those with Parkinson's disease or vascular dementia, indicating that CD8^+^ T cells may be involved in the pathogenesis of AD (Pirttilä et al., 1992; Lombardi et al., 1999). CD8^+^ T cell infiltration is closely related to p-tau accumulation in human AD patients and mouse models (Merlini et al., 2018; Stojić-Vukanić et al., 2020). The decrease in the percentage of circulating CD8^+^ T cells may be attributed to the fact that these cells are recruited into the brain from the periphery, leading to AD progression in the central nervous system. CD8^+^ T cells are located near microglia and are involved in neuronal processes both in patients and in animal models (Merlini et al., 2018; Unger et al., 2018; Gate et al., 2020; Stojić-Vukanić et al., 2020). The production of B lymphocytes decreases with age (Stephan et al., 1998). Our analysis showed a more evident decrease in B lymphocytes in AD patients than in age-matched HCs, suggesting that ongoing AD may exacerbate aging characteristics.

The decrease in hemoglobin levels in patients with AD can be explained by multiple influencing factors. Decreased hemoglobin level in the blood reduces cerebral blood perfusion, resulting in neuroinflammation- and oxidative stress-mediated production of brain Aβ (Babiloni et al., 2014; Salminen et al., 2017; Raz et al., 2019), which exacerbates neurodegenerative diseases. Weiss et al. found that the RDW level was elevated by inflammation through iron metabolism impairment, inhibition of proliferation of erythroid progenitor cells, and release of immature RBCs (Weiss and Goodnough, 2005). The RDW is strongly associated with circulating inflammatory markers, such as high-sensitivity C-reactive protein, and erythrocyte sedimentation rate (Lippi et al., 2009). Decreased RBC counts and hemoglobin levels and increased RDW and MCV were found in AD and MCI patients in some studies (Chang et al., 2007; Öztürk et al., 2013; Du et al., 2020). However, no statistical evidence of association was shown in our analysis of RBC counts, RDW, or MCV, except for the significantly decreased hemoglobin level in AD patients compared to HCs. This result may be attributed to the fact that the number of included relevant studies on these indices was small, while there were as many as 10 studies for the meta-analysis of hemoglobin.

Similar to the results of previous studies, there was no statistically significant change in PLT counts or MPV values in patients with AD or MCI in this meta-analysis. We found significantly reduced PDW levels in patients with AD compared with HCs. Some studies have reported that the number of coated platelets, a subset of activated platelets, is positively associated with AD progression (Prodan et al., 2007, 2008). Jaremo et al. showed that densities of platelets were significantly different in patients with AD and age-matched HCs (Järemo et al., 2012). Thus, platelet heterogeneity may account for inconsistent results. The reason for the significantly reduced PDW in AD is unclear. PDW can be used to indicate variations in platelet size and differentiate thrombocytopenia categories (Borkataky et al., 2009). The cytokines and chemokines released by abnormal platelet activation may be involved in AD pathogenesis through neuroinflammation, such as macrophage inflammatory protein-1a, RANTES (Iarlori et al., 2005), platelet endothelial cell adhesion molecule-1, and intercellular adhesion molecule-1 (Nielsen et al., 2007). Future studies on the association of AD-related dementia with different types of PLTs, relevant indices (MPV and PDW), and cytokines are needed.

Some of the peripheral blood indices, such as the NLR, MPV, and PDW, have been explored as diagnostic biomarkers for AD. Dong et al. reported an NLR cutoff point of 2.35 for differentiating patients with AD from HCs, with a sensitivity and specificity of 83 and 54%, respectively (Dong et al., 2019). Kuyumcu et al. established a similar NLR cutoff of 2.48, with a sensitivity and specificity of 70 and 80%, respectively (Kuyumcu et al., 2012). Similar but lower sensitivities and specificities were determined for differentiating MCI patients from HCs (An et al., 2019; Dong et al., 2019). Owing to the limited number of studies using receiver operator characteristic curves, we could not perform a meta-analysis to explore the value of these indices as diagnostic biomarkers.

The limitations of our meta-analysis are as follows: First, heterogeneity was identified in several comparisons, possibly because of varying ages or different stages of AD among the studies. Stratified analysis could not be performed because of insufficient and inconsistent stage criteria for AD. Second, all included studies were cross-sectional and inevitably subject to selection bias. The duration of AD, treatment of AD, and combined chronic diseases were confounding factors. Third, few studies have explored the differences between AD and MCI patients, so that there was not enough data to perform a meta-analysis for any peripheral blood cell or index between AD and MCI. Fourth, the results of comparisons between MCI and HCs were not so convincing due to the limited numbers of studies (2–4 studies) included for them. To clarify the association between the severity of AD-related dementia and peripheral inflammatory and immune alterations, well-designed studies are needed in future.

## Conclusion

Our work supports the theory that peripheral inflammatory and immune profiles are associated with AD. According to our data, patients with AD had significantly increased leukocyte and neutrophil counts, increased NLR and CD4/CD8 ratio, as well as decreased lymphocyte counts, hemoglobin levels, PDW, and percentage of CD4^+^ T cells and B lymphocytes. Furthermore, significantly elevated neutrophil and leukocyte counts and NLR were observed in MCI patients compared with HCs. Future studies should focus on the diagnostic value of these peripheral blood cells and indices for AD-related dementia.

## Data Availability Statement

The original contributions presented in the study are included in the article/Supplementary Materials, further inquiries can be directed to the corresponding authors.

## Author Contributions

L-TH, Y-BW, and J-HW conceived and designed the study. L-TH and J-HW took full responsibility for data collecting, performed the meta-analysis, systematic review, and drafted the manuscript. C-PZ, Y-BW, and J-HW helped revise the manuscript. All authors have read and approved the final manuscript.

## Funding

This work was funded by National Key R&D Program of China (Nos. 2018YFC2002100 and 2018YFC2002103).

## Conflict of Interest

The authors declare that the research was conducted in the absence of any commercial or financial relationships that could be construed as a potential conflict of interest.

## Publisher's Note

All claims expressed in this article are solely those of the authors and do not necessarily represent those of their affiliated organizations, or those of the publisher, the editors and the reviewers. Any product that may be evaluated in this article, or claim that may be made by its manufacturer, is not guaranteed or endorsed by the publisher.
