# Supplementary material for: Association of Peripheral Blood Cell Profile With Alzheimer's Disease: A Meta-Analysis
Source: Front Aging Neurosci. 2022 May 6;14:888946. doi: 10.3389/fnagi.2022.888946 (PMC9120416; doi:10.3389/fnagi.2022.888946)
Supplement: Supplementary file 1 [file Data_Sheet_1.PDF]

Supplementary Figures. Sensitive analysis for each peripheral blood cell comparison between Alzheimer's disease patients and healthy controls. Each study corresponds to a circle and a horizontal line. The circle represents the pooled estimate of the other studies after omitting the given named study. The length of the horizontal line represents the 95% confidence interval.

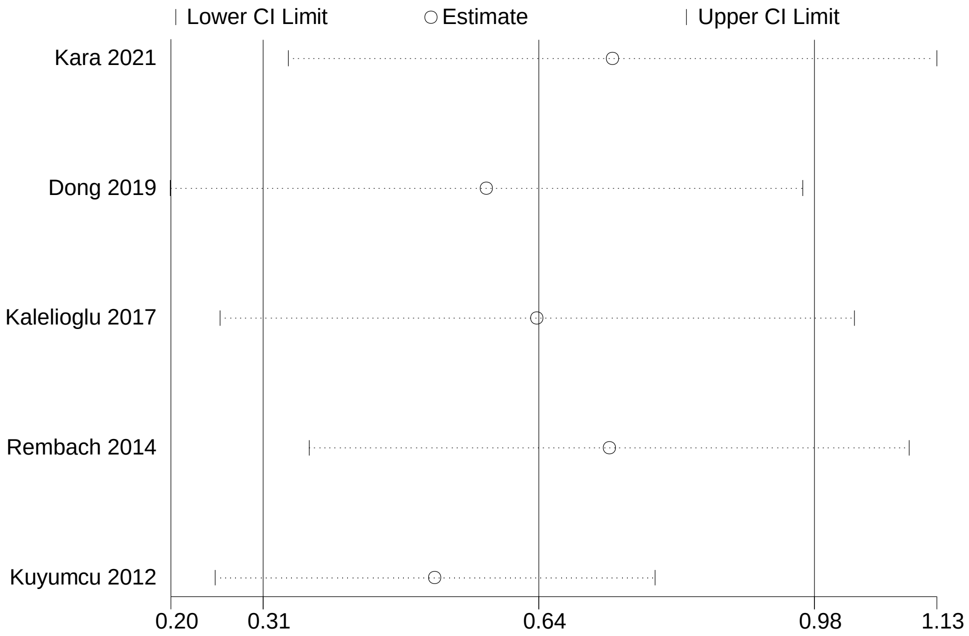

Supplementary Figure 1. Sensitive analysis for neutrophil–lymphocyte ratio comparison between Alzheimer's disease patients and healthy controls.

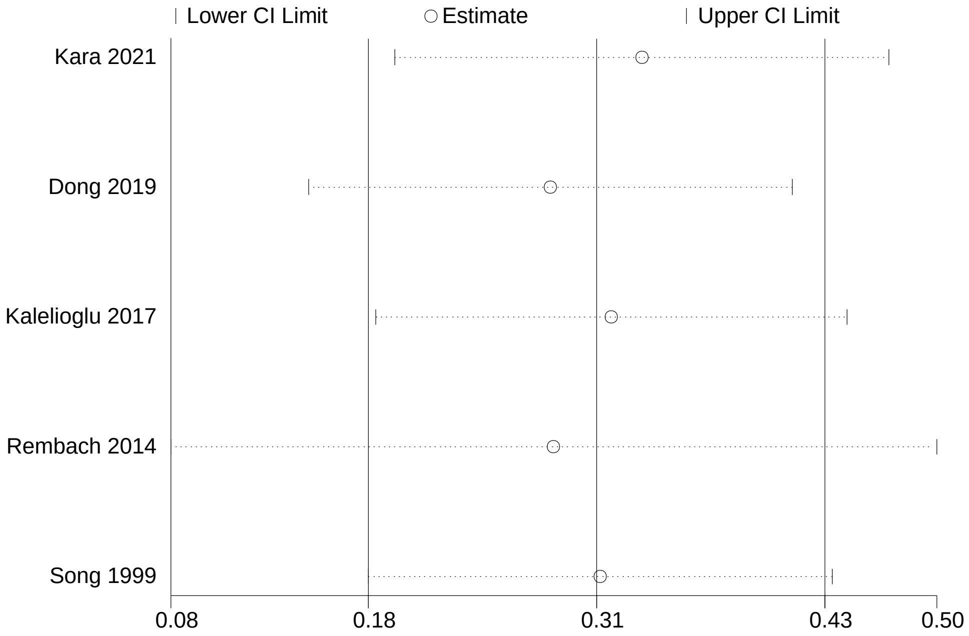

Supplementary Figure 2. Sensitive analysis for neutrophil counts comparison between Alzheimer's disease patients and healthy controls.

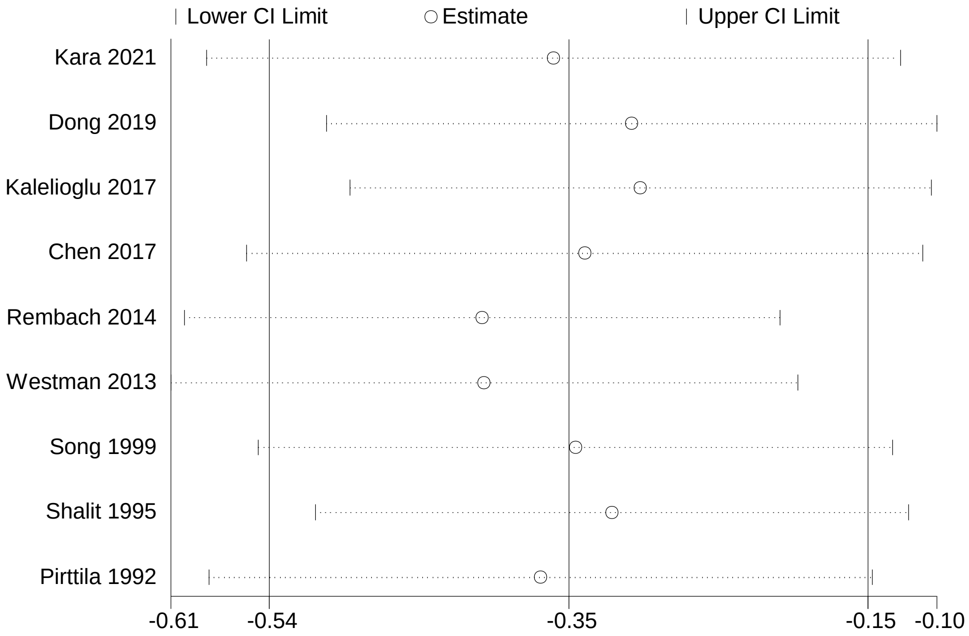

Supplementary Figure 3. Sensitive analysis for lymphocyte counts comparison between Alzheimer's disease patients and healthy controls.

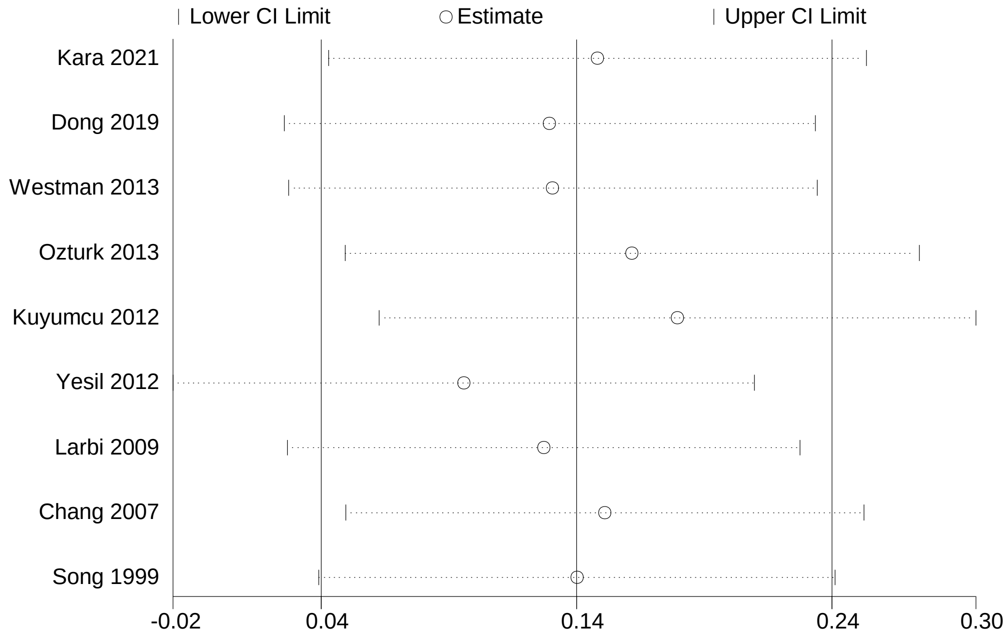

Supplementary Figure 4. Sensitive analysis for leucocyte counts comparison between Alzheimer's disease patients and healthy controls.

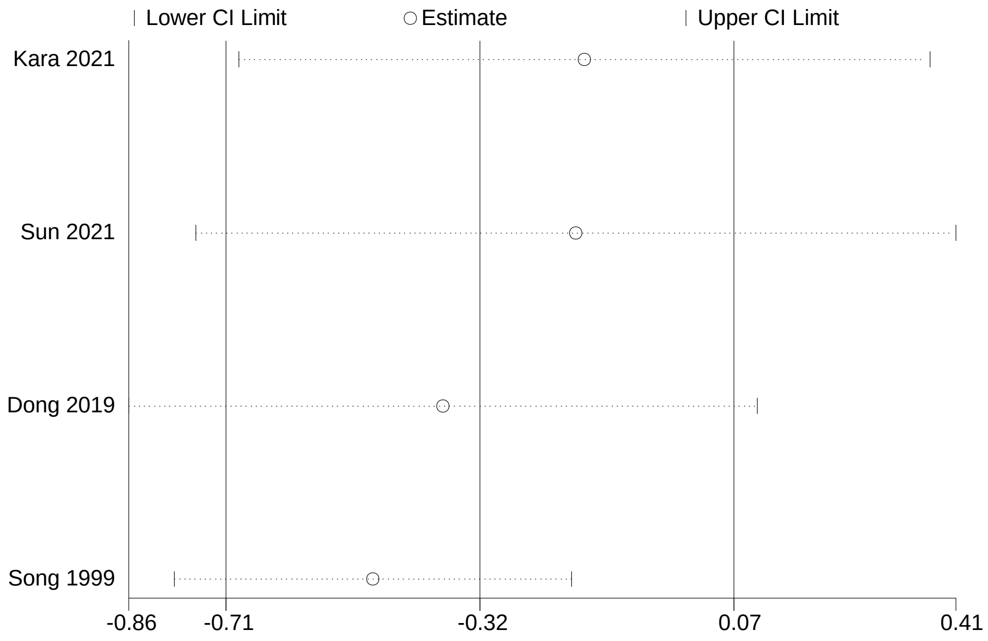

Supplementary Figure 5. Sensitive analysis for monocyte counts comparison between Alzheimer's disease patients and healthy controls.

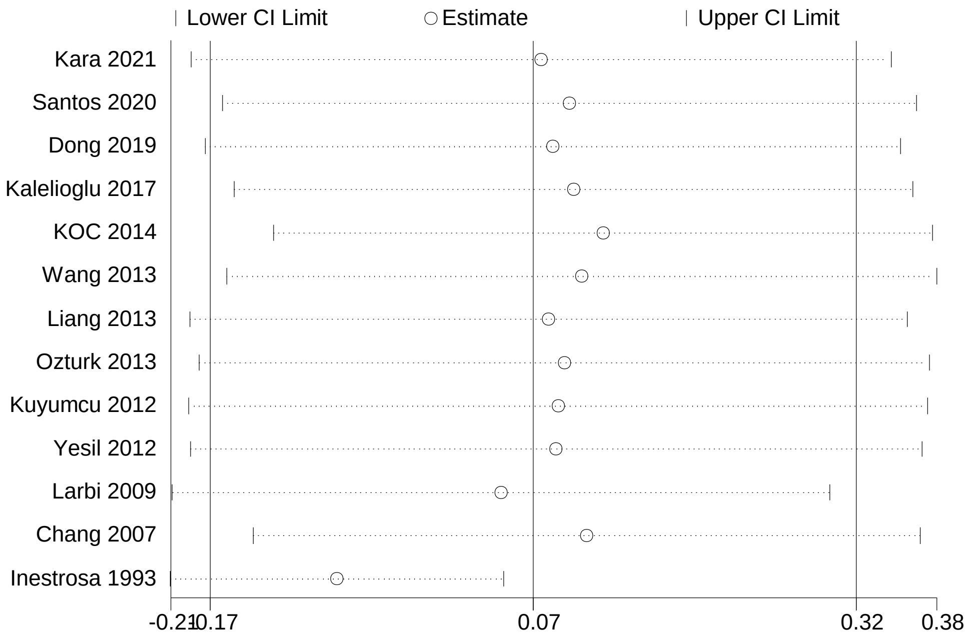

Supplementary Figure 6. Sensitive analysis for platelet counts comparison between Alzheimer's disease patients and healthy controls.

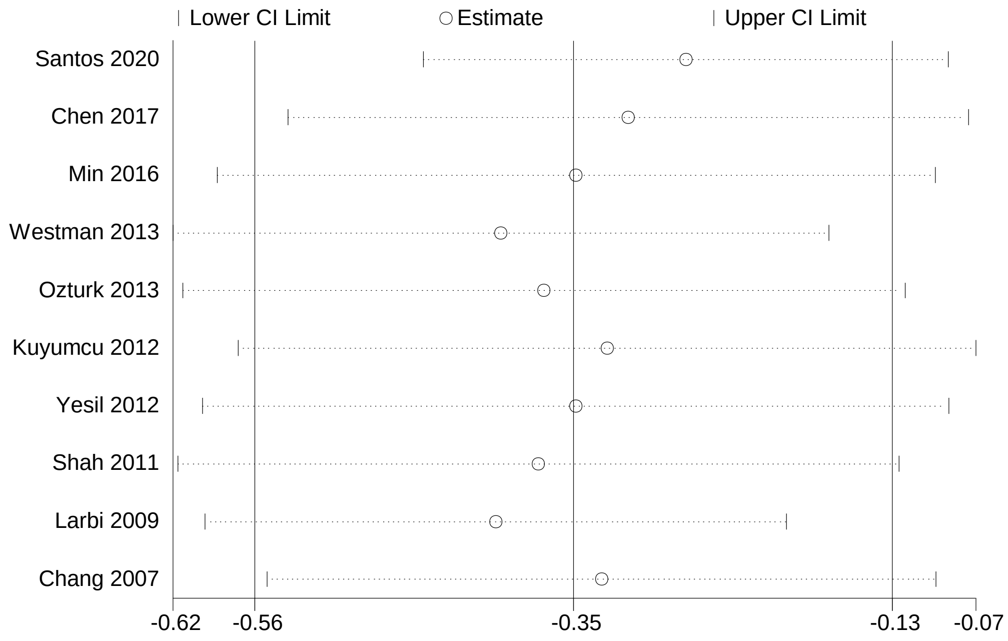

Supplementary Figure 7. Sensitive analysis for hemoglobin comparison between Alzheimer's disease patients and healthy controls.

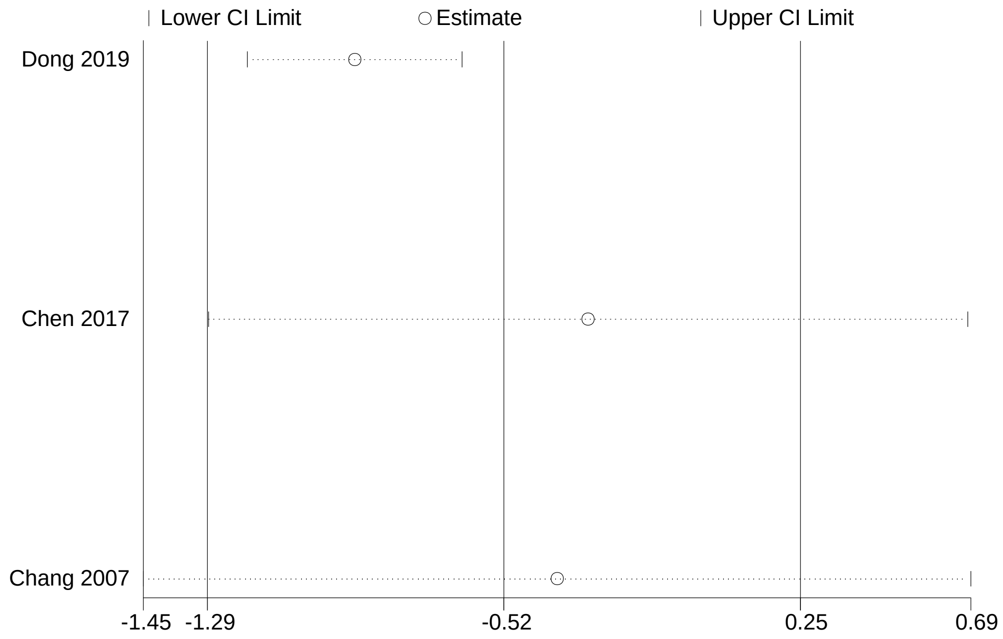

Supplementary Figure 8. Sensitive analysis for red blood cell counts comparison between Alzheimer's disease patients and healthy controls.

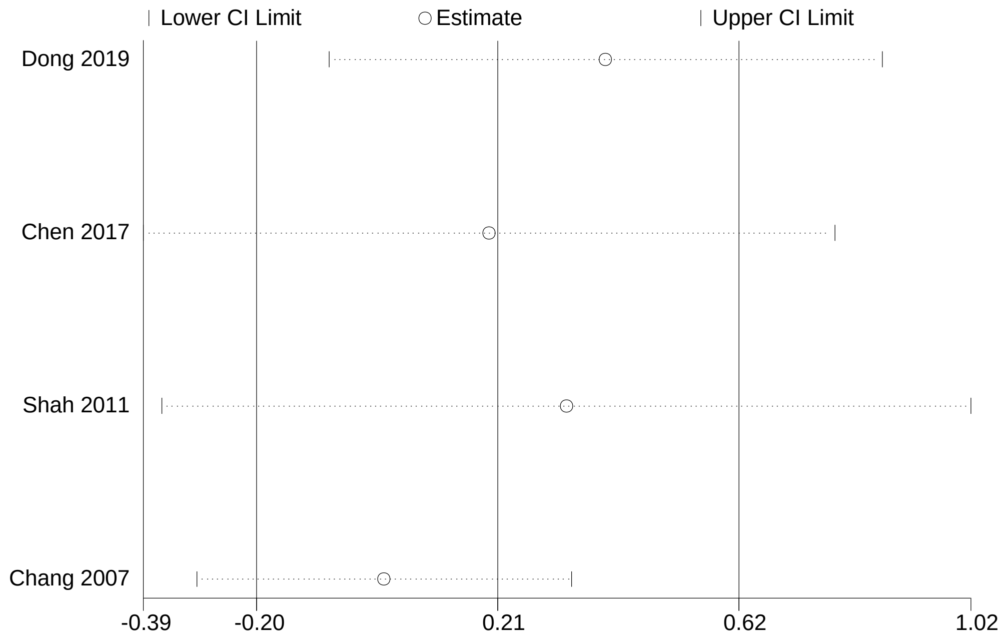

Supplementary Figure 9. Sensitive analysis for mean corpuscular volume comparison between Alzheimer's disease patients and healthy controls.

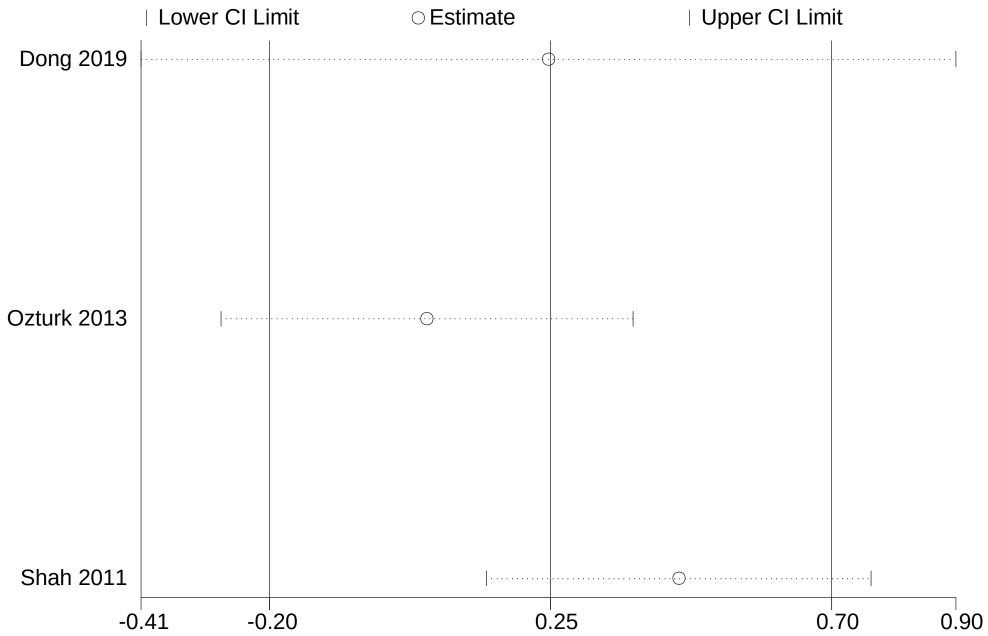

Supplementary Figure 10. Sensitive analysis for red cell distribution width comparison between Alzheimer's disease patients and healthy controls.

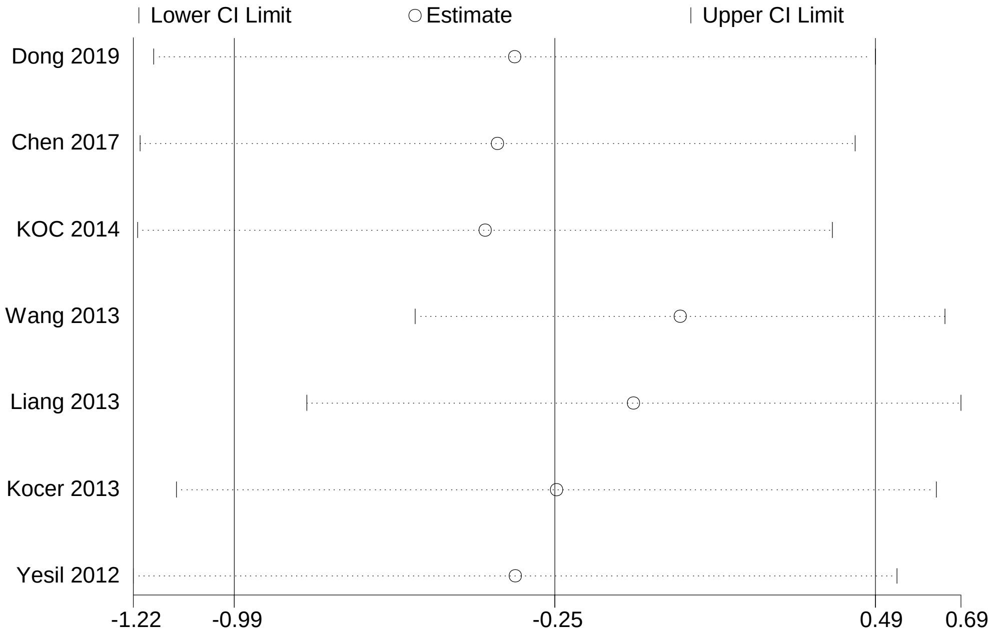

Supplementary Figure 11. Sensitive analysis for mean platelet volume comparison between Alzheimer's disease patients and healthy controls.

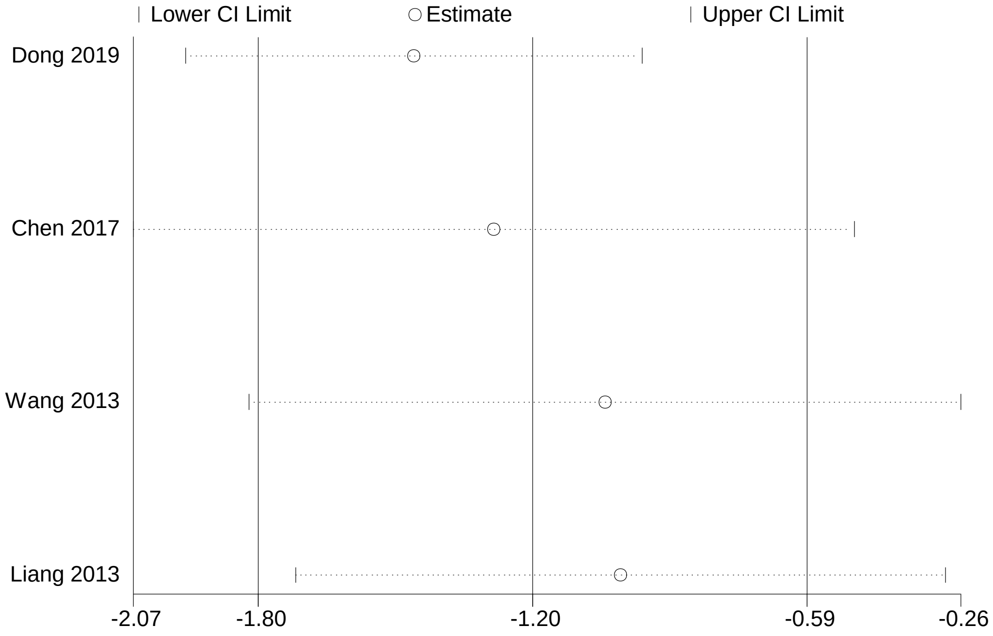

Supplementary Figure 12. Sensitive analysis for platelet distribution width between Alzheimer's disease patients and healthy controls.

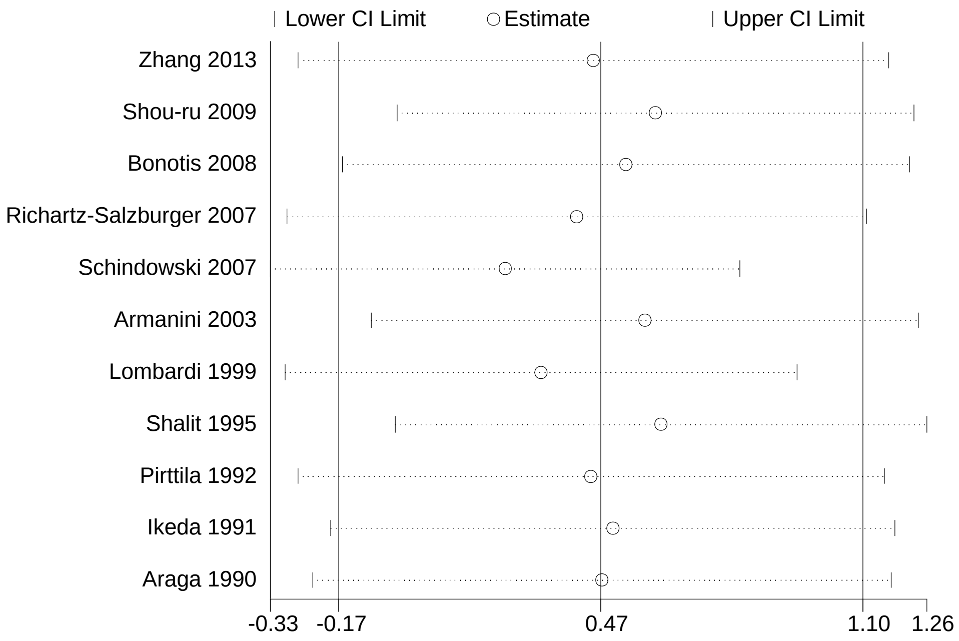

Supplementary Figure 13. Sensitive analysis for CD4^{+} T cell percentage between Alzheimer's disease patients and healthy controls.

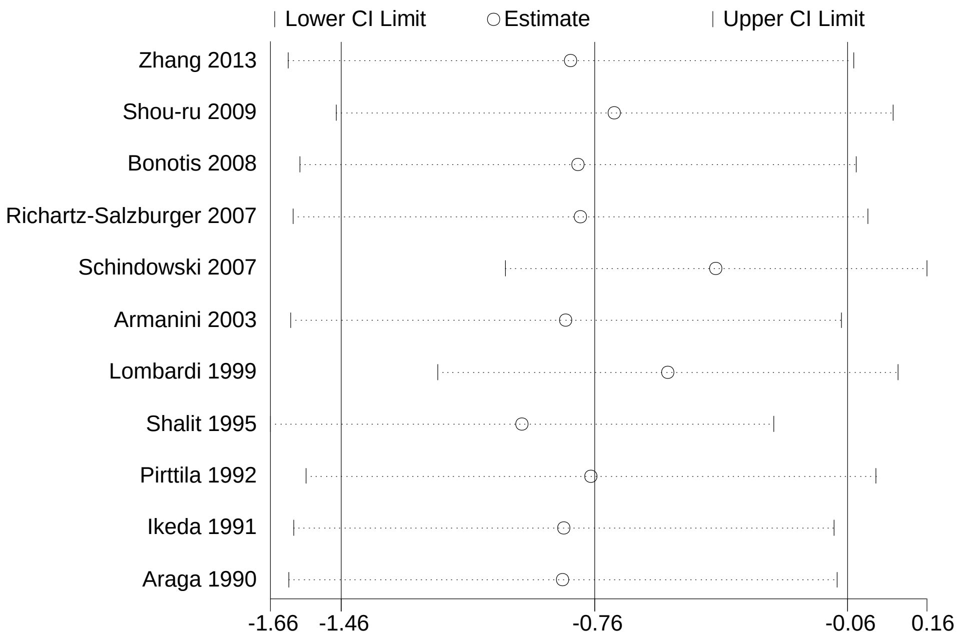

Supplementary Figure 14. Sensitive analysis for CD8^{+} T cell percentage between Alzheimer's disease patients and healthy controls.

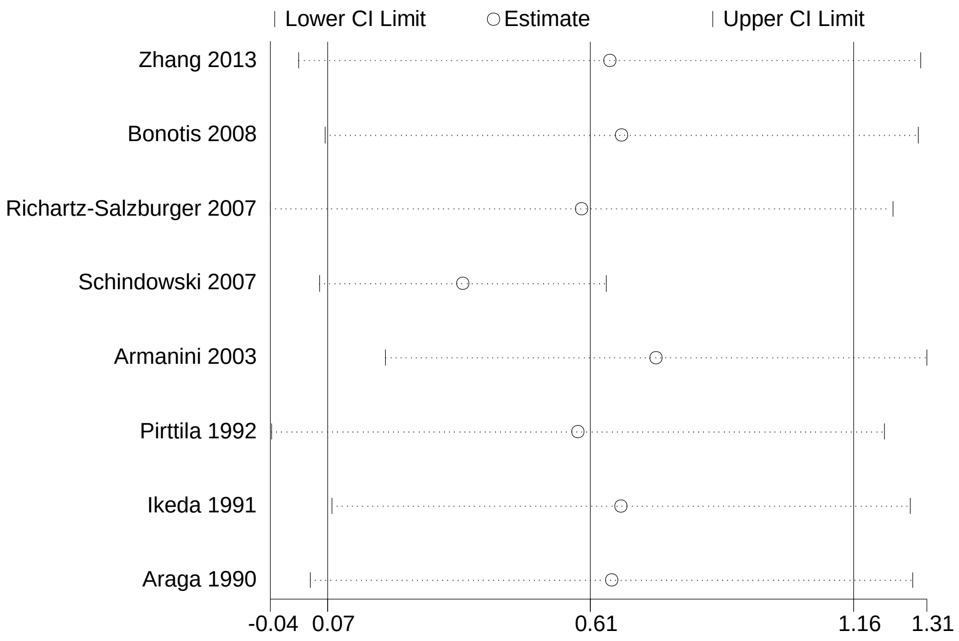

Supplementary Figure 15. Sensitive analysis for CD4/CD8 ratio between Alzheimer's disease patients and healthy controls.

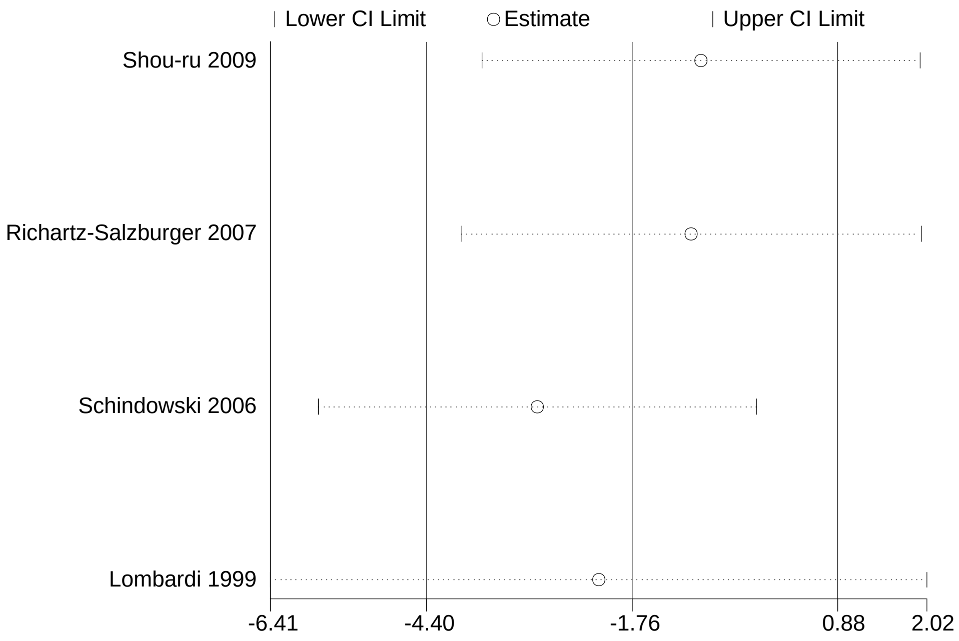

Supplementary Figure 16. Sensitive analysis for $CD3^{+}$ T cell percentage between Alzheimer's disease patients and healthy controls.

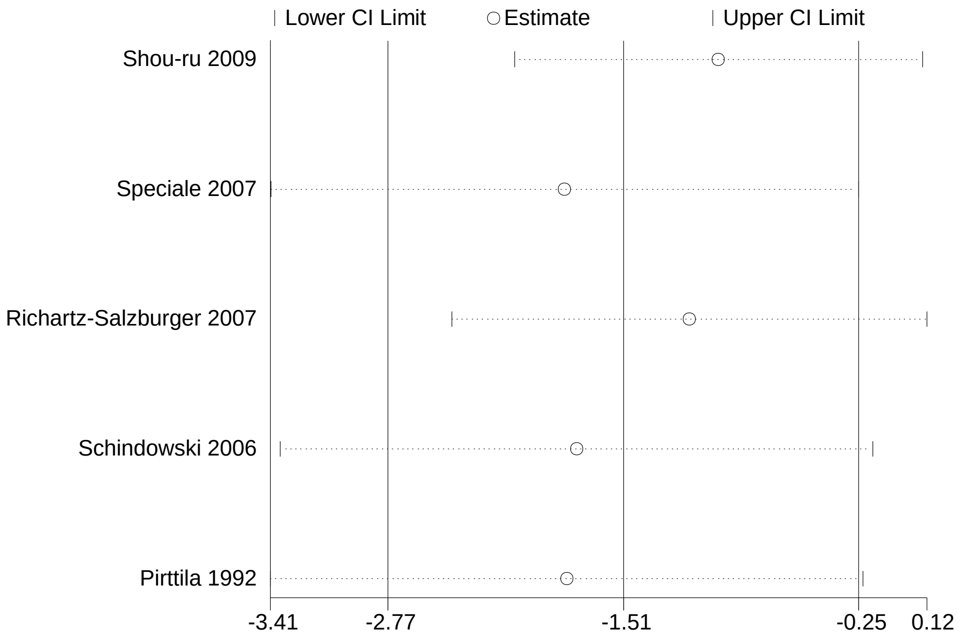

Supplementary Figure 17. Sensitive analysis for B lymphocyte percentage between Alzheimer's disease patients and healthy controls.

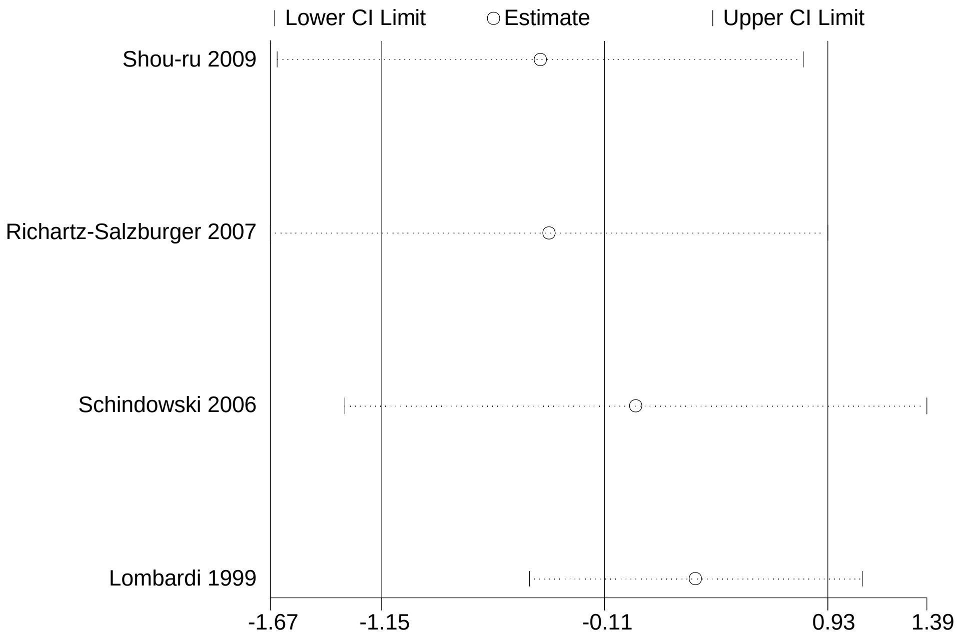

Supplementary Figure 18. Sensitive analysis for natural killer cell percentage between Alzheimer's disease patients and healthy controls.
